# Samba II PCR testing for COVID-19 in pregnant women: a retrospective cohort study and literature review

**DOI:** 10.1186/s12884-021-03653-4

**Published:** 2021-03-17

**Authors:** Ruiling Xu, Tara Alicia Pauley, Hannah Missfelder-Lobos, Richard John Haddon, Ravindra Kumar Gupta, Hsu Phern Chong

**Affiliations:** 1 Department of Obstetrics & Gynaecology, Rosie Maternity Hospital, Cambridge, CB2 0SQ UK; 2 Department of Anaesthesia, Cambridge University NHS Hospitals Foundation Trust, Cambridge, CB2 0QQ UK; 3 Department of Medicine, University of Cambridge, Cambridge, CB2 0AW UK

**Keywords:** COVID-19, SARS-CoV-2, Pregnancy, Universal screening

## Abstract

**Background:**

Asymptomatic carriage of COVID-19 in pregnant women has been reported and could lead to outbreaks in maternity units. We sought to ascertain the impact of rapid isothernal nucleic acid based testing for COVID-19 in an unselected cohort of pregnant women attending our maternity unit. We also assessed the correlation between community prevalence and asymptomatic carriage.

**Methods:**

Data for the retrospective cohort study were collected from a large UK tertiary maternity unit over a 4-week period using computerised hospital records. Literature searches were performed across multiple repositories. COVID-19 prevalence was extracted from online repositories.

**Results:**

Nasopharyngeal and oropharyngeal swabs were obtained from 457/465 (98%) women during the study period. The median turnaround time for results was 5.3 h (interquartile range (IQR) 2.6–8.9 h), with 92% of the results returned within 24 h. In our cohort, only one woman tested positive, giving a screen positive rate of 0.22% (1/457; 95% CI: 0.04–1.23%). One woman who tested negative developed a fever postnatally following discharge but was lost to follow-up. From our literature review, we did not find any correlation between asymptomatic carriage in pregnant women and the reported regional prevalence of COVID-19.

**Conclusions:**

Testing using the SAMBA-II machine was acceptable to the vast majority of pregnant women requiring admission and had a low turnaround time. Asymptomatic carriage is low, but not correlated to community prevalence rates. Screening pregnant women on admission will remain an important component in order to minimise nosocomial infection.

**Supplementary Information:**

The online version contains supplementary material available at 10.1186/s12884-021-03653-4.

## Background

The World Health Organization (WHO) characterized Coronavirus disease 2019 (COVID-19) as a global pandemic caused by severe acute respiratory syndrome coronavirus 2 (SARS-CoV-2) in March 2020 [1]. The first case series was described by Huang et al. [2] where 41 patients were admitted to Jin Yin-Tan Hospital in Wuhan, China. Of the 41 confirmed cases, 98% had fever and 76% had a dry cough, all developed pneumonia. Deep sequencing of lower respiratory tract samples identified the viral genome to be from the coronavirus family and this was named (COVID-19). Cellular access is gained through a spike protein that binds to the angiotensin-converting enzyme 2 receptor (ACE2), and viral uptake is promoted by the type 2 transmembrane serine protease (TMPRSS2) [3]. Lauer reported a median 5.7 days to fever onset (CI 4.9–6.8 days), and 97.5% of cases had a fever within 12.5 days (CI 8.2–17.7 days of exposure) [4]. Importantly, COVID-19 often takes a prolonged disease course with viral load in throat and nose declining after the first week as antibody titres rise [5]. This can result in nucleic acid testing becoming negative after the first week, though neutralisation and SARS-CoV-2 antibodies are readily detectable in nearly all cases [6]. Asymptomatic carriage of COVID-19 was estimated to be 15% in general population [7] and Sutton et al. [8] reported that positive rate of 13.7% (29/210) among asymptomatic group and 87.9% (29/33) of pregnant women infected with SARS-CoV-2 were asymptomatic at presentation.

Identification of asymptomatic pregnant women with COVID-19 is important for several reasons. Firstly, identification of asymptomatic carriers would allow changes in the pathway of care so as to prevent nosocomial infections, reducing the risk of asymptomatic transmission to other pregnant women and also to healthcare workers (HCW). Testing also enables early isolation and rationalisation of personal protective equipment (PPE). Thirdly, identification of viral carriage could allow closer monitoring both during and after delivery, consideration of low molecular heparin for prophylaxis of venous thromboembolism, and longer term follow-up.

SAMBA II is an isothermal point of care nucleic acid amplification based platform with a detection limit of around 250 genome copies/ml [9]. It has previously been clinically validated in parallel with standard reverse transcription polymerase chain reaction (RT-PCR) with sensitivity and specificity of 96.9% (95% CI 84.2–99.9) and 100% (95% CI 96.9–100) for COVID-19, respectively. The median time to result has been significantly reduced from 26.4 h (IQR 21.4 to 31.4) for the standard lab RT-PCR test to 2.6 h (IQR 2.3 to 4.8) for SAMBA II SARS- CoV-2 test [10].

The aims of this paper are two-fold: to ascertain the incidence of asymptomatic carriage in our maternity unit using a rapid diagnostic testing platform; and to assess if the rate of carriage of COVID-19 in asymptomatic pregnant women correlated with peak local prevalence.

## Methods

### Study design and setting

Cambridge University Hospitals NHS Foundation Trust (CUH) covers a large geographical area with a total population of approximately 5 million people in the east of England. The Rosie Maternity Hospital is part of CUH, with an annual delivery rate of approximately 5500 women. All admissions to the Rosie Maternity Hospital were offered a nasopharyngeal (NP) and oropharyngeal (OP) swab for COVID-19. These included women who were required antenatal and postnatal admission to the obstetric wards, women attending the main delivery unit and birth centre, and women attending for elective Caesarean Section or cerclage insertion. All staff members received training prior to obtaining swabs. Samples were processed using the SAMBA II machine (Diagnostics for the Real World, Chesterford, UK) [11, 12]. Data were extracted for population demographics and symptomatology. Symptoms were defined as fever and or cough in line with Public Health England’s advice at the time. Patients who were asymptomatic were defined as women who lacked a fever and/or cough from the time of testing to discharge. Turnaround time was defined as the time the swab was collected, to the time a result was uploaded onto the electronic hospital record. Where time entries were missing, electronic hospital notes were retrieved to check for a record of sample collection and availability of results. These were computed separately. Our study was registered as a service evaluation project and ethical approval was not required.

### Data and literature review

We conducted a literature search from PubMed, Cochrane COVID-19 trials for published studies, MedRxiv for pre-prints and an unofficial online repository on 15/05/2020 and updated on 07/08/2020. Where datasets from the same institution were replicated in more than one publication, but with a larger sample size or longer duration, the publication with the largest sample size and/or duration was selected. Data were extracted for case definition, diagnostic test employed, duration of follow up, gestational age at the time of testing and turnaround time. In order to assess if the prevalence of COVID-19 positive symptomatic and asymptomatic pregnant women correlated with local prevalence rates, we extracted local population COVID-19 prevalence rates for each of the included studies from open source repositories (Supplementary information).

### Statistical analysis

Data were analysed using GraphPad Prism version 5.00 for Windows, GraphPad Software, San Diego California USA, www.graphpad.com. Data were extracted by two reviewers (RX and HC), and disagreements were resolved by discussion with a 3rd reviewer (TP).

## Results

### SAMBA-II results

During the period 07/05–06/06 (4 weeks) 465 women attended the Rosie Maternity Hospital for obstetric indications. NP and OP swabs were obtained from 457/465 women upon admission (98%). 18 (3.84%) women had more than one swab taken either due to prolonged inpatient admission beyond 7 days, or reattendance within the 4 week period. Self-reported ethnicity was available for 99.5% of women. The majority of our population consisted of White British, Irish or European ancestery (407/465, 87.5%) and this is reflective of the local population in Cambridgeshire. The median gestational age at the time of the admission swab was 39 weeks (Interquartile range (IQR) 37–40 weeks). Six women (1.3%) attended in the postnatal period due to obstetric concerns. The median duration of inpatient stay for all patients was two days (IQR 1–3). The median duration of follow-up in the post-natal period was 5 days (IQR 3–9).

None of the women had a cough on admission. 37/465 women developed a fever either during labour or following delivery (7.9%). All 37 women had a negative result on admission, and the fever was attributed to an obstetric cause. Two women were admitted with breathlessness on a background of cardiac disease. One woman developed symptoms of fever or sore throat four days after delivery. Advice from Public Health England at the time was to contact a national helpline and she was lost to follow up.

Only one woman in our cohort tested positive over this four-week interval. This woman had neither cough nor fever on admission, but had symptoms of altered taste and smell four weeks prior to her admission. To ensure that this was a true positive, NP and OP swabs were repeated using the SAMBA II machine (but not included in our current analyses for TAT) until she tested negative. As this patient remained symptom-free during admission, she would be the only true asymptomatic COVID-19, giving an asymptomatic carriage rate of 0.22% (1/457; 95% CI: 0.04–1.23%). Knowledge of her results enabled HCW to convert from using standard surgical masks, plastic aprons and gloves to full personal protective equipment when caring for this patient.

**Fig. 1:**
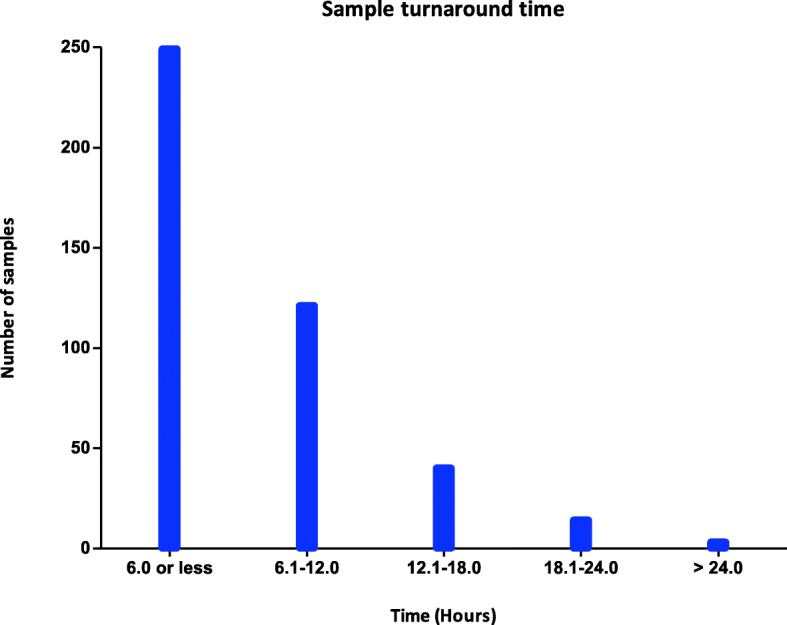
Results turnaround times (TAT) from 432 SARS-CoV-2 testing samples, using SAMBA II for in-hospital testing

Result turnaround times (TAT) were verified and available for 432/457 (94.5%) samples. The median TAT was 5.3 h (IQR 2.6–8.9) (Fig. 1), with 93.9% (429/457) of sample results returned within 24 h. Where sample turnaround times were absent, these were due to an underestimation of turnaround times rather than a delay in sample processing. This information was gleaned from verbatim entries in the patient records regarding the sample obtained, and the results entered. As no specific record of time was entered, these were excluded from further analyses. A small percentage (16/465, 3.4%) of women declined to be swabbed on admission, 5 of them were in discomfort from labour therefore declined testing. No reasons were given for the remaining 11 women who declined testing.

### Literature review

Four hundred and seven articles were screened for title and abstract and 46 full-text articles assessed for inclusion. 17 universal screening studies [13–29] (Table 1) were selected to assess if the prevalence of COVID-19 positive pregnant women correlated with local prevalence rates as these papers fulfilled our prespecified criteria above.

**Table 1:** Community COVID-19 prevalence and asymptomatic carriage in pregnant women

Author	Region/Country	Type of study	Peak daily case load at start of the study period for relevant area	Max daily case load during study period for relevant area	Peak daily case load the end of the study period for relevant area	Number of pregnant women in study	Number tested positive	Number asymptomatic at the time of admission AND tested positive	Test positivity %	Proportion of asymptomatic test positive %
Fassett	California, USA	Observational retrospective study	1261	1921	1921	3923	17	17	0.43	100.00
Gagliardi	North of Tuscany and Liguria, Italy	Case series	5210	6153	4053	533	3	2	0.56	66.67
Herraiz	Madrid, Spain	Observational retrospective study	6278	6740	2610	203	2	1	0.99	50.00
Naqvi	California, USA	Case series	1104	1331	1268	82	1	0	1.22	0.00
LaCourse	Washington, USA	Retrospective cohort	2	437	314	188	5	1	2.66	20.00
Ceulemans	North East Flanders, Belgium	Case series	683	2319	426	470	13	8	2.77	61.54
Miller	Illinois, USA	Case series	1150	2023	2023	635	23	10	3.62	43.48
Ochiai	Tokyo, Japan	Retrospective analysis	383	743	203	52	2	2	3.85	100.00
Campbell	Connecticut, USA	Case series	377	1100	620	770	30	22	3.90	73.33
Khalil	London, UK	Cohort study	822	1071	434	129	9	8	6.98	88.89
Yassa	Istanbul, Turkey	Prospective cohort	2131	2936	1035	296	23	12	7.77	52.17
Prabhu	New York, USA	Prospective cohort	2166	9909	6693	675	70	55	10.37	78.57
Doria	Senhora da Hora, Portugal	Case series	302	1516	514	103	12	11	11.65	91.67
Sutton	New York, USA	Cohort study	2166	8775	8775	215	33	29	15.40	87.60
Buckley	New York, USA	Case series	9073	9909	8064	307	50	50	16.29	100.00
Vintzileos	New York, USA	Retrospective cohort	6555	9909	9410	161	32	21	19.88	65.63
London	New York, USA	Retrospective cohort	73	9909	8064	156	68	22	43.58	32.35

**Fig. 2:**
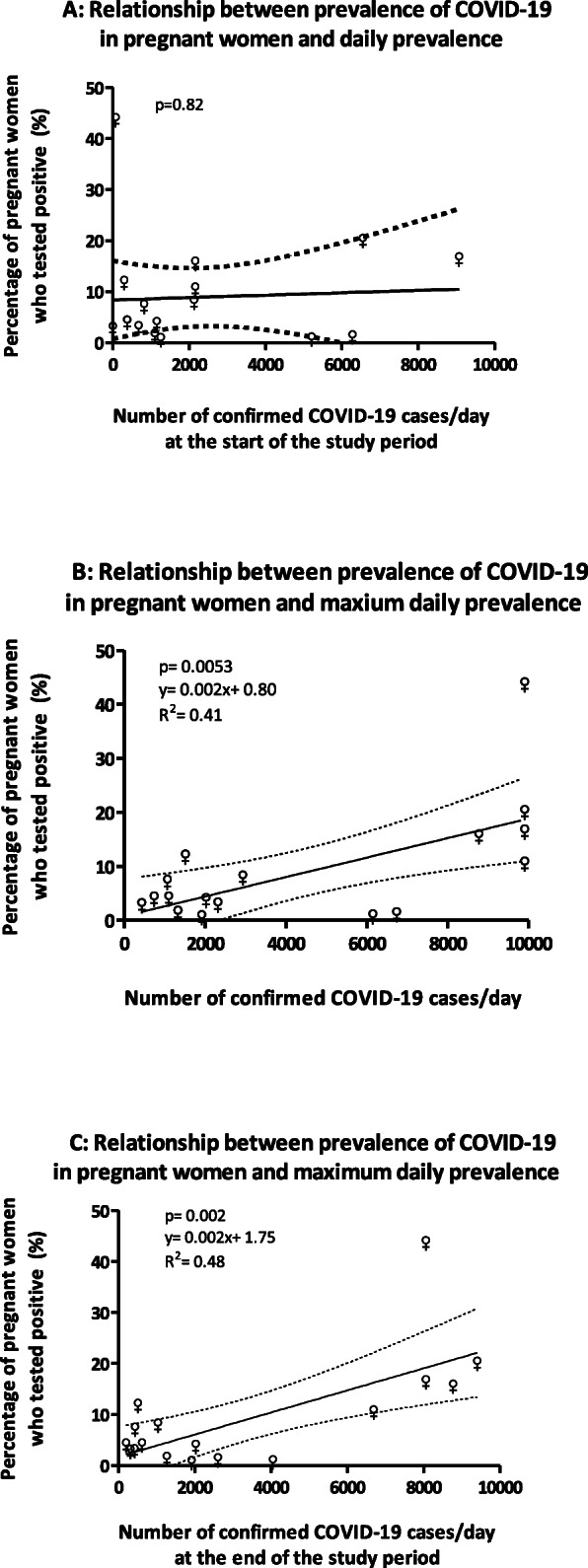
Relationship between prevalence of COVID-19 in pregnant women and published peak prevalence. Data from Table 1 was used to generate Figure 2. Simple linear regression was performed to investigate the relationship between the prevalence of COVID-19 in pregnant women and peak daily community prevalence at three time points: the start of the study interval the maximum reported daily prevalence and the prevalence at the end of the study interval. The scatter plots demonstrated a positive correlation with the maximum reported daily prevalence rates during the study interval (R^2^ = 0.41, *p* = 0.0053) and towards the end of the study period (R^2^ = 0.48, *p* = 0.002) but not with rates at the start (R^2^ = 0.004, *p* = 0.82)

**Fig. 3:**
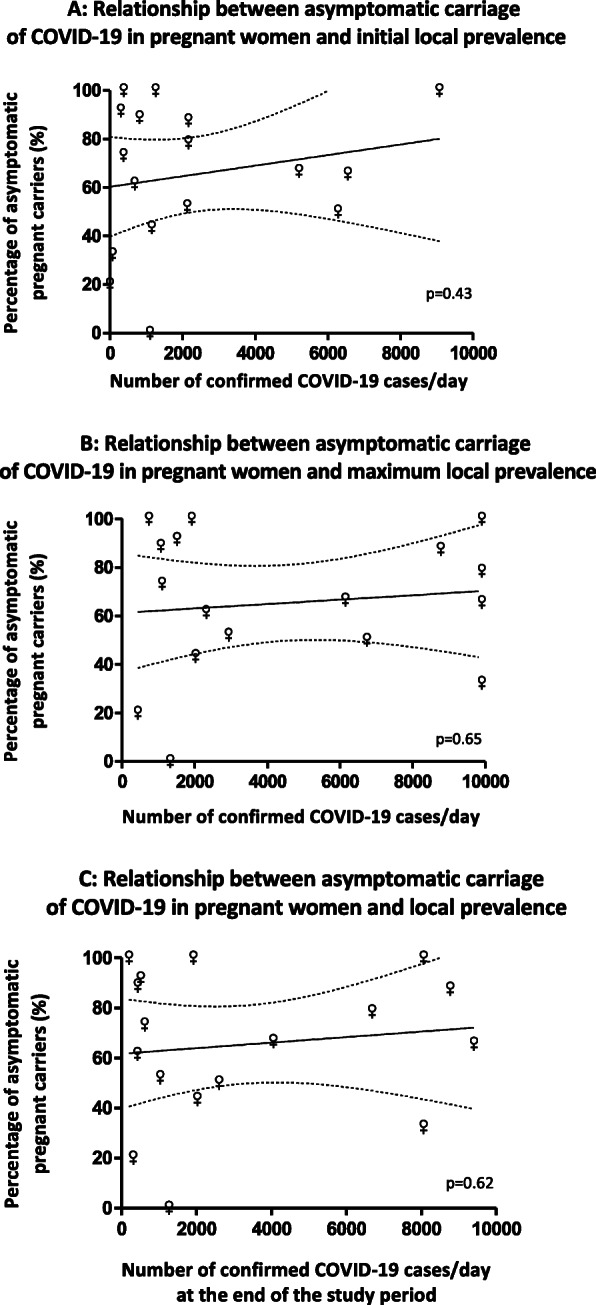
Relationship between asymptomatic carriage of COVID-19 in pregnant women and local peak prevalence. Data from Table 1 was used to generate Figure 3. Simple linear regression was performed to investigate the relationship between the prevalence of COVID-19 in pregnant women and peak daily community prevalence at three time points: the start of the study interval, during and end of the study interval. The scatter plots demonstrated no correlation with the maximum reported daily prevalence rates at the start (R^2^ = 0.04, *p* = 0.44), during (R^2^ = 0.01, *p* = 0.65) and end of the study intervals (R^2^ = 0.02, *p* = 0.62)

Data presented are in ascending order of the total proportion of pregnant women who tested positive. Data for peak daily reported COVID-19 prevalence at the start, during and end of the relevant study periods were retrieved from internationally published repositories [30–32]. This data was used to generate Figs. 2 and 3. NP- Nasopharyngeal, OP- Oropharyngeal

We found a correlation between the test positivity with the regional background prevalence rates of COVID-19. There was a direct correlation between the number of confirmed cases at the peak (R^2^ = 0.41, *p* = 0.0053) (Fig. 2b) and end of the study period with those who tested positive (R^2^ = 0.48, *p* = 0.002) (Fig. 2c), but not with the number of confirmed cases at the start of the study period (R^2^ = 0.004, *p* = 0.82) (Fig. 2a). There was no correlation between the proportions of asymptomatic pregnant carriers with background infection rates (Fig. 3).

Only three papers reported turnaround time. Breslin et al. [33] reported an average of 8 h for 43 women screened [Conventional PCR, (New York, New York)] and LaCourse et al. [17] reported a median turnaround time of 2.5 h for rapid testing [DiaSorin Simplexa (MDX Liaison) EUA assay, *n* = 82 women] and 7.1 h for routine PCR (Seattle, Washington). London et al. [29] reported an average turnaround time of 5 h using GeneXpert PCR in a cohort of 75 women (Brooklyn, New York).

## Discussion

### Main findings

Our work summarises existing literature on universal screening of asymptomatic carriage of COVID-19 in pregnant women with specific reference to the turnaround time of results, and local prevalence rates. The key findings of our study are threefold. We captured data on all attendances in a large UK maternity hospital and ours is the largest, to have reported turnaround times of less than 24 h in over 90% of samples returned. We were also able to demonstrate a high acceptability of COVID-19 screening amongst our maternity population with only 3% declining to be tested. Thirdly, ours is the first study to demonstrate the utility of point of care testing in an unselected cohort of pregnant women attending a large UK maternity unit.

Whilst the data are reflective of our maternity service, laboratory services were also processing samples from patients attending the accident and emergency department, and medical and surgical wards within a national health service. We were still able to achieve a short turnaround time of five hours. This allowed for changes to be made within postnatal care pathway, and escalation of the use of personal protective equipment. Testing of newborns could have also been performed had it been required.

The low positive screening rate in our cohort may be partly explained by the low rates of infection in the east of England (cumulative infection rate 0.42%), in comparison to 1.27% in California and 2.12% in New York. As the pandemic evolved, advice and guidance were issued to pregnant women in the UK, who may have adopted behavioural changes that minimised interaction with the general public. Additionally, home working and minimising the commute to work could have protected them further. Interestingly, the proportion of asymptomatic carriers did not correlate with the regional infection rate. Possible explanations include variations in mask wearing, local test and trace strategies, movement control orders and specific advice on “shielding” in pregnant women [34].

Many hospitals introduced visiting restrictions during the lockdown, and ours was no different. Partners were not allowed to attend the antenatal ward nor ultrasound scan appointments. Obstetric and non-obstetric face-to-face clinic appointments were changed to phone appointments where possible, thus reducing footfall within the hospital premises. Staff working in clinical areas were required to wear personal protective equipment in the form of a surgical mask, gloves and aprons as a minimum, and FFP3 masks for aerosol generating procedures. Thus, pregnant women attending hospital should also be reassured, not only that asymptomatic carriage of COVID-19 is low, infection within the inpatient setting is low. Whether or not this is maintained against a backdrop of rising COVID-19 infections rates is unknown.

### Strengths and limitations

We did not compare the test accuracy of the Samba-II machine in our population. However, this device has been tested previously in a cohort of over 1000 individuals and found to have 97% accuracy to conventional RT-PCR testing [10]. There is no reason to believe why it should perform differently in pregnant women. In contrast, GeneXpert PCR testing has been previously been validated on a much smaller cohort of less than 50 women [35]. We did not perform radiological investigations to look for manifestations of COVID-19 pneumonia, as some others have clinical diagnosis criteria as well as laboratory diagnosis [36, 37]. Women could therefore have a negative NP or OP result for COVID-19, but have radiological changes. However, the likelihood of this is low. Although we had a high acceptability rate, we did not explore women’s and staff views on screening for COVID-19, thus further research is required to evaluate this. For example, there may be a need to develop tests which are acceptable to women in labour, or training for hospital staff in contact tracing where women simply declined to be tested. In the interest of patient safety, we would suggest a conservative approach to patient pathways for women who decline testing so as not to result in contagion within the hospital setting.

We accept that anosmia and lack of taste can be considered a symptom of COVID-19 infection. However, we used pre-specified criteria for asymptomatic screening to ensure consistency in case definition so as not to exclude women screened prior to 18/5/2020, when changes in symptom definition were published [38]. Indeed, Allotey et al. have subsequently demonstrated that only 5% of asymptomatic pregnant and recently pregnant women with COVID-19 exhibit altered sense of taste [34]. Given that only one woman tested positive in our cohort, inclusion of anosmia and or ageuisia as a symptom would not alter our results as her symptoms had resolved by the time she was tested. As the Samba-II process is not based on cycle time we are unable to comment on viral load for this patient. However Celik et al. have demonstrated that Covid-19 viral shedding from the upper respiratory tract has a mean duration of 17 days (up to a maximum of 83 days) but is not infectious beyond day 9 of illness [39].

A potential limitation of SAMBA II testing relates to financial implications as the machine costs £35,000 with test capsules costing £35 each. The financial cost implications of asymptomatic screening within maternity has not been evaluated. However, given the high infectivity of COVID-19, asymptomatic screening within a hospital setting will remain an important component so as to avoid nosocomial infection, prevent patient-to-staff infection, and to rationalise the use of full personal protective equipment. Another disadvantage is testing capacity, given that each machine can only handle only one test at a time, and each run lasting 1.5 h. Our unit has mitigated this by running 20 machines simultaneously. Additional barriers have been manufacturing enough machines, reagents and cartridges for testing. Nonetheless SAMBA II has been procured by NHS England and is being implemented in over 100 sites nationally.

## Conclusion

The Royal College for Obstetricians and Gynaecologists in the UK have recently published guidelines on testing for asymptomatic pregnant women attending maternity units [40]. These are broadly in line with our current practice but for the type of swab being offered (conventional lab based RT-PCR as opposed to rapid testing with the SAMBA II). Owing to the variable rates of asymptomatic carriage of COVID-19 in pregnant women, and the rapid turnaround time using the SAMBA II machine, we propose the introduction of SAMBA II or other point of care testing platforms in maternity units as an accurate and acceptable test to pregnant women requiring admission.

## Supplementary Information

**Additional file 1.**

## Data Availability

The datasets generated for the cohort study are not publically available due to current UK regulations with regards to data protection, but are available from the corresponding author on reasonable request. The datasets generated for the peak coronavirus prevalence rates can be found from international repositories listed in the references.

## Abbreviations

NP: Nasopharyngeal
OP: Oropharyngeal
CUH: Cambridge University Hospitals NHS Foundation Trust
COVID-19: Coronavirus-19
TAT: Results turnaround time
RT-PCR: Real time quantitative fluorescence polymerase chain reaction
IQR: Interquartile range
